# Capturing diversity and cultural drivers of food choice in eastern India

**DOI:** 10.1016/j.ijgfs.2020.100249

**Published:** 2020-12

**Authors:** Arindam Samaddar, Rosa Paula Cuevas, Marie Claire Custodio, Jhoanne Ynion, Anindita Ray (Chakravarti), Suva Kanta Mohanty, Matty Demont

**Affiliations:** a International Rice Research Institute, Los Baños, Laguna, Philippines; b Department of Food & Nutrition, Maharani Kasiswari College, University of Calcutta, India; c Kalinga Institute of Industrial Technology, KIIT School of Management, Bhubaneswar, India; d Institute of Rural Management Anand (IRMA), Anand, Gujrat, India

**Keywords:** Gastronomic systems research, Expert elicitation, Food choice, Nutrition, Eastern India

## Abstract

The EAT-Lancet Commission urgently called for “planetary health diets”. The success of encouraging dietary shifts, however, crucially hinges on people, and more specifically on consumers' culture, context, socioeconomic status, food environment, attitudes, perceptions, beliefs, and behavior towards food choice. In India, enhanced food availability and accessibility do not readily lead to improved nutritional status. Thus, developing planetary health diets in India requires an understanding of systemic drivers of food choice. Food is an essential part of Indian culture and deeply rooted to the country's history, traditions, lifestyles, and customs. Yet, the diversity and cultural drivers of food choice are still insufficiently understood. To address this knowledge gap, we use expert elicitation to contextualize the “gastronomic systems research” framework to a target population of low-to middle-income households to capture the diversity and cultural drivers of food choice and its nutritional implications in rice-based diets in two states in eastern India. The experts catalogued 131 unique dishes associated with five differentiated daily dining occasions. The majority of dishes belong to the starch food group. Morning snacks exhibit the lowest nutritional diversity while dinners feature the highest diversity in both states. In West Bengal, dish options tend to be carbohydrate-rich and energy-dense, and a significant number of dishes are fried and oily. The gastronomic system mapped by the experts provides a useful baseline for nutritionists, policymakers, and food system actors as a first step in the design of nutrition intervention strategies to develop planetary health diets in eastern India.

## Introduction

The year 2019 was pivotal for the science of food choice. First, the EAT-Lancet Commission, a non-profit think-tank of 37 leading scientists from 16 countries in various disciplines including human health, agriculture, political sciences and environmental sustainability, published an urgent call for “planetary health diets”; i.e., diets that improve both human health and environmental sustainability (Willett et al., 2019a). Planetary health diets are seen as the core vehicle for attaining the United Nations’ Sustainable Development Goals and the Paris Agreement by 2050. These diets, low in animal-sourced food and composed mainly of plant-based food sources, require radically transforming global food systems through major paradigm shifts in dietary habits and diets. The success of adopting such planetary health diets, however, crucially hinges on people, and more specifically on their culture, context, socioeconomic status, food environment, attitudes, perceptions, beliefs, and behavior towards food choice (Cuevas et al., 2021). Yet, the diversity and cultural drivers of food choice are still insufficiently understood. Second, in 2019 the Nobel Prize was awarded to prominent scholars in the field of behavioral economics for the second time in history after Vernon L. Smith and Daniel Kahneman in 2002. The 2019 Laureates Esther Duflo, Abhijit Banerjee, and Michael Kremer demonstrated through various studies the crucial role of human behavior in food subsidies and nutritional and health intervention programs in developing countries. Banerjee and Duflo (2007), for example, emphasized that the poor maximize pleasure, not necessarily nutrition. The design of food security policies often naively assumes that if food is subsidized, people will automatically think of nutrition; i.e., policies typically do not account for hedonic drivers of food choice. Haddad (2020) similarly echoes the urgent need to invest in the science of food choice: “The private sector spends billions of dollars on influencing consumers to buy certain foods and influencing policymakers to shape the regulatory infrastructure. The public sector spends very little on understanding why people consume the foods they do and on why decision makers take the decisions they do. We need much more consumer insight research into the former and more political economy work into the latter.”

**Fig. 1:**
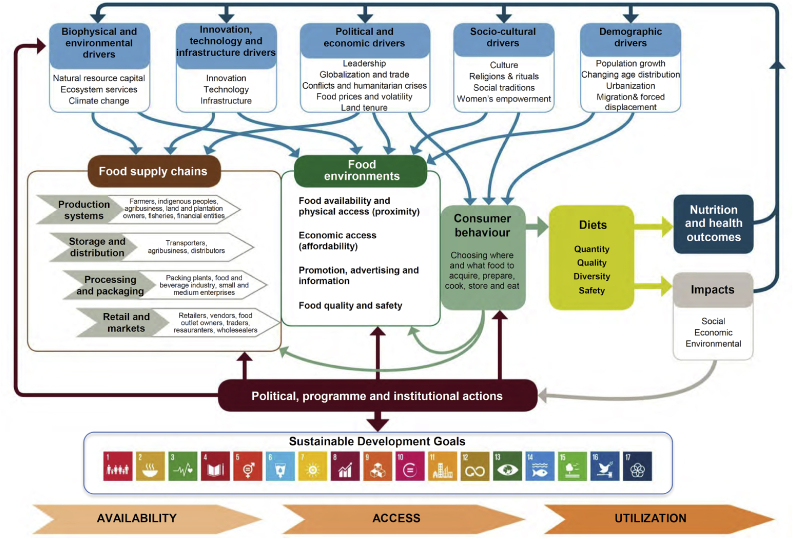
Conceptual framework of food systems for diets and nutrition. Source: HLPE (2017).

**Fig. 2:**
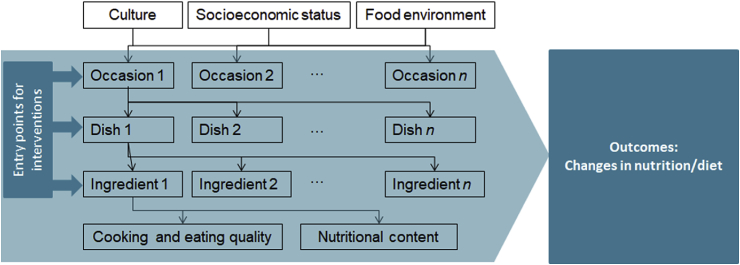
The Gastronomic Systems Research (GSR) framework. Source: Cuevas et al. (2021); adapted from Cuevas et al. (2017).

As concluded by the 2019 Nobel Prize Laureates, people do not just eat nutrients or ingredients; instead, dietary patterns are part of a system, which is shaped and driven by culture, context, socioeconomic status, food environment, and hedonic motivation (Cuevas et al., 2021). The High Level Panel of Experts on Food Security and Nutrition of the Committee on World Food Security (HLPE, 2017) recently proposed a holistic, conceptual framework for food systems for diets and nutrition (Fig. 1). The framework explicitly recognizes the role of culture as a driver of food environments and consumer food choice behavior and diets, the latter being presented as the critical link between food supply chains and nutrition and health outcomes. The academic community has realized that generating a better understanding of cultural and hedonic drivers of food choice requires merging the fields of gastronomy and food science, exemplified by Elsevier's launch of the *International Journal of Gastronomy and Food Science* in 2012 and forthcoming book *Gastronomy and Food Science* in 2020. In response to this need for interdisciplinary and systemic approaches, Cuevas et al. (2017, 2021) recently proposed the Gastronomic^2^ Systems Research (GSR) framework (Fig. 2) in these publication outlets, arguing that the cultural drivers of food choice are shaped by a hierarchical system of food consumption occasions or meals that command a set of dish options, which, on their turn, determine a set of ingredients and ingredient pairings that have particular hedonic qualities and nutritional attributes, and feature specific cooking methods. Consumption of these dishes results—ultimately—in intermediate outcomes such as nutrition diversity of consumer diets, and final outcomes such as nutrition and health status of consumers. Thus, the GSR framework not only helps the science of food choice gain a better understanding of why, when, and what people eat; it also unveils possible entry points for nutrition interventions that can assist nutritionists, policymakers, and food system actors in developing planetary health diets.

Two of India's poorest states, Odisha and West Bengal (both in eastern India), are the perfect venues to study the science of food choice through the GSR framework with the aim of formulating nutrition interventions to nudge consumers towards planetary health diets. In Odisha, 32.6% of the population lives below the poverty level (Thomas et al., 2015). In West Bengal, around 20% of the population lives below the national poverty threshold (Ministry of Statistics and Program Implementation, 2014). In both states, people mainly eat starchy staples (e.g., rice and potato), which provide energy but are nutritionally poor (M. Banerjee et al., 2013; Panda et al., 2015). More nutritious food (e.g., milk and vegetables) is reported to be largely inaccessible to the poor (P. K. Bhattacharya, 2013; Chand and Gartia, 2016). Even with people's dependence on starchy staples, gender-disaggregated state- and community-based measures of chronic energy deficiency indicate that significant proportions of the populations in both Odisha and West Bengal are undernourished (Das and Bose, 2015; Singh and Mukherjee, 2015). The high level of food insecurity is associated with high mortality rates in Odisha (Rahman, 2016). On the other hand, undernutrition has been linked with widespread stunting and thinness in adolescents in West Bengal (Pal et al., 2017).

Achieving planetary health diets in eastern India will be impossible, though, without a baseline on the diversity of food choices and a thorough understanding of the systemic cultural drivers of food choice. Food is an essential part of Indian culture and deeply rooted to the country's history, traditions, lifestyles, and customs (Sen, 2004). In fact, the foundation of various traditional dishes prepared and eaten by people in India is derived from “Ayurveda”, an Indian traditional medicine system based on the human body as the product of food intake (Pant, 2010; Sarkar et al., 2015). Previous studies have indicated that Indian consumers are increasingly considering health benefits in food choices (Birol et al., 2010; Landes and Gulati, 2004; Pingali and Khwaja, 2004). It is also indicated that consumers in middle- and higher-income groups are significantly upgrading and diversifying their diets by including higher valued food items (Ali et al., 2010; Brokaw and Lakshman, 1995; Khush, 2005; Landes and Gulati, 2004; Pingali and Khwaja, 2004). However, information is scant about poor people's preferences, nutritional intake, and awareness about various food items they consume on a regular basis. In eastern India, for instance, fragmented studies report effects of malnutrition in the region (e.g., India State-Level Disease Burden Initiative Malnutrition Collaborators, 2019; Prost et al., 2019) but the nutritional intake and interventions required to improve it have not been investigated comprehensively.

Eating behaviors are often associated with caloric intake and body mass metrics. Yet, increasing food availability (i.e., productivity) and consumer accessibility (i.e., increasing consumer income) does not automatically translate into improved nutritional status (Verhart, van den Wijngaart, Dhamankar and Danielsen, 2015), particularly in India (FAO, 2010; FAO, IFAD, & WFP, 2015). Investing in improving nutrition is crucial in accelerating economic development through increased productivity and poverty reduction (World Bank, 2006). Little is known about how eating behaviors drive food choices (Falguera et al., 2012; French et al., 2012) but food choice is an important consideration in developing nutrition interventions. It must be noted that food habits are shaped by the processes of choosing, acquiring, distributing, cooking, serving, and sharing food (Almerico, 2014; Kittler et al., 2012). On the other hand, food choices can also serve as a means of communicating personal identity and emotional reasoning (i.e., food voice); for example, refusing to partake of a certain food (e.g., veganism or fasting as a means of political protest) or opting for specific dishes can deliver a powerful message (Hauck-Lawson, 2004). In addition, food choices tell the story of family struggles, migrations, resistance, assimilation, adaptation, and group identity. Food choice results from a complex set of human behaviors and is influenced by biological and genetic influences on eating behavior as well as socioeconomic and cultural factors (Arganini et al., 2012; Grimm and Steinle, 2011) and is not only about consuming calories for sustenance. Thus, any attempt in developing interventions to improve the nutritional status of consumers has to consider systemic drivers of food choice such as culture, context, socioeconomic status, food environment, and hedonic motivation.

The GSR framework has been previously piloted in the Philippines and applied to the case of rice-based diets of a target population of middle-to high-income Filipino consumers (Cuevas et al., 2017). However, the framework has not yet been implemented in the socioeconomic context of poor households and the rich cultural and gastronomic context of India. The current paper aims to address the research gaps mentioned throughout this introduction and provide a useful baseline for nutritionists, policy makers and food system actors as a first step in the design of nutrition intervention strategies to develop planetary health diets in eastern India. To achieve this goal, we use expert elicitation to contextualize the GSR framework to a target population of low-to middle-income households in Odisha and West Bengal to capture the diversity and cultural drivers of food choice and its nutritional implications in rice-based diets in eastern India.

The paper is organized as follows. After this introduction, in Section 2 we detail our methodology for capturing diversity of food choice through expert elicitation following the GSR framework. Section 3 presents and discusses the results, and Section 4 concludes and identifies areas for future research.

## Methodology

### Contextualizing the GSR framework through expert elicitation

Cuevas et al. (2021) recommend contextualizing the GSR framework through expert elicitation as a first methodological step in the qualitative-quantitative research continuum. Identifying the components of the different levels of the GSR framework is best captured by consulting professional experts in a structured way as a means to access limited data with insufficient information. An expert elicitation workshop is a cost-efficient and rapid appraisal method to identify the gastronomic system of a target population. The identification and selection of experts is crucial to ensure the validity and quality of results. A snowball approach enabled the team to start with a large network of interdisciplinary experts referred by Chairs of Nutrition Departments and by NGOs ensuring the diversity in our sample of experts from the public and private sector (Kirchherr and Charles, 2018). Therefore, we targeted experts from four different professional backgrounds: Nutrition, Home Science, Food Technology, and Foodservice Industry (i.e., chefs and restaurateurs). To mitigate possible sampling bias due to the non-random nature of this method, the team met with various stakeholders, conducted repeated call-backs, and follow-up of respondent referrals (Kirchherr and Charles, 2018). Face-to-face meetings with the identified experts and selection criteria were used to finalize the list of experts, such as expertise with the subject, willingness to collaborate, experience with research and overall knowledge about the State in terms of food consumption and culture across different regions and urban/rural populations. The inclusion of restaurateurs and chefs as representatives of the food service industry further anchored our findings in the real world where consumer behavior and food choices manifest. Several authors have proposed collaboration frameworks between chefs and scientists to study the interaction between gastronomy and food science (e.g., Frøst, 2019; Fooladi et al., 2019).

We conducted two expert elicitation workshops in eastern India, i.e. in Kolkata, West Bengal and in Bhubaneswar, Odisha, July 18 and 21, 2017. The basic approach of the expert elicitation process was to facilitate system-level thinking, active engagement in individual- and group-level discussions, and to encourage out-of-the box thinking and consensus building. The methodology is described in more detail in the accompanying *Data in Brief* article (Custodio et al., 2020).

**Fig. 3:**
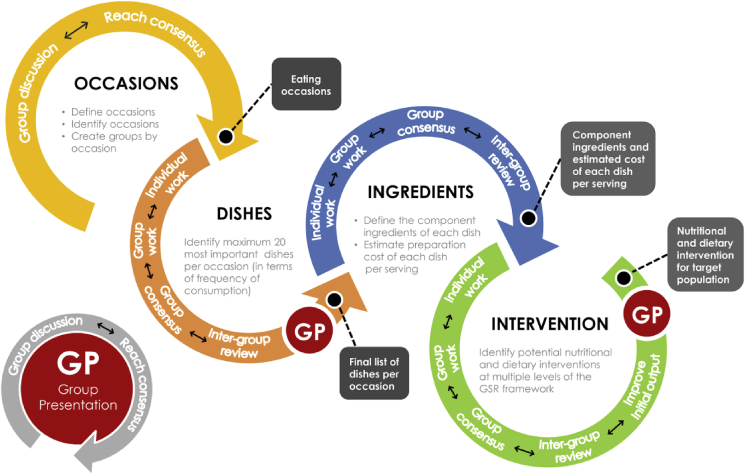
The structure and process of expert elicitation (EE). The EE is composed of four major exercises targeting different components of the GSR framework: (i) occasions, (ii) dishes, (iii) ingredients, (iv) intervention. The double-headed arrow illustrates the exchange of ideas among expert participants during the exercise which eventually generates an output at the end, which is shown in each of the grey boxes. Illustration: Neale Paguirigan (IRRI).

Experts were first requested to define the target population and were then introduced to the GSR framework (Cuevas et al., 2021). Experts followed a standard structure of individual, intra-team, and inter-team elicitation methods (Fig. 3) to identify the gastronomic system for their state in terms of eating occasions, dishes, and ingredients. The experts were given the renowned Indian cookbook written by Prof. Pushpesh Pant (2010) as reference material to check and validate dishes and verify their recipes and ingredients. The entire gastronomic system for each state was captured in a database which provides a baseline of current diversity and cultural drivers of food choice in eastern India (Ynion et al., 2020).

Finally, the experts were requested to review the gastronomic system they constructed and identify and rank the top three most urgent nutrition interventions that can improve the baseline of rice-based diets they identified in each state. For each intervention, they specified (i) the nutritional concern; (ii) the target population; (iii) the entry point of the intervention in the GSR framework (occasions, dishes or ingredients); (iv) the action or intervention that needs to take place to address the nutritional concern of the target population; (v) the agents of change that are responsible for the action or intervention; and (vi) the impact pathway responsible for generating the improved nutritional outcomes. This exercise provided a last chance to revisit the gastronomic system and also served as a validation to check whether the experts had sufficiently understood the multi-layered and hierarchical structure of the GSR framework.

The GSR framework (Fig. 2) is embedded in the HLPE (2017) framework as it unravels the interaction between (i) demographic, socio-cultural, political and economic drivers; (ii) the food environment; (iii) consumer behavior; and (iv) consumer diets (Fig. 1). First, the drivers are captured through the choice of our target population, i.e. low-to middle-income households in urban and rural eastern India; a different choice of target population would result in a different gastronomic system. Within a given culture, the gastronomic system is not homogenous among all socio-economic classes. Clearly, economic factors such as income largely influence the type of food (premium or basic) and the occasions (e.g., dining out, special occasions, etc.) when food is consumed (Mottaleb et al., 2018a, 2018b). Second, consumer behavior and diets are assessed by the experts by identifying the gastronomic system of the target population in terms of occasions, dishes, and ingredients, given the typical food environments to which the households are exposed. The food environment is implicitly captured during the mapping of the gastronomic system and is considered as a separate factor in the GSR framework (Fig. 2). The food environment can determine the gastronomic system adopted by a household, irrespective of the culture and socio-economic status; for example, an immigrant family is dependent on what the food environment has to offer. According to Downs et al. (2020), the sociocultural and political environment (e.g., education, policies, governance, national income, religion, culture) and ecosystem influence the food environment. Hence, in the GSR framework (Fig. 2), economic factors and education are subsumed and captured in “Socioeconomic status” and “Food environment.”

The expert elicitation is the first step in a research continuum that captures food choice in an increasingly rigorous way with increasing granularity. While providing a baseline of diversity and cultural drivers of food choice in the gastronomic system, the expert elicitation needs to be followed-up with research that can capture actual consumer behavior, such as (i) consumer surveys to validate the information obtained from the experts and estimate frequencies of the occurrence of eating occasions, dishes and ingredient pairings (Cuevas et al., 2017), as well as to capture the heterogeneity of food environments that individual households may face (e.g., urban versus rural); and (ii) behavioral experiments to elicit and nudge food choice behavior or behavioral intentions (Demont et al., 2019).

### Nutritional analysis

To zoom in further on the nutritional implications of the rice-based diets identified during the expert elicitation, one of the two states was selected for further in-depth study, based on the available expertise. The state of West Bengal was selected and the expert from the Department of Food & Nutrition, Maharani Kasiswari College, Calcutta University was subsequently invited to become a co-author of this study and assigned the task of refining the list of dishes captured by the experts in the Kolkata expert elicitation and conduct nutritional analysis. The methodology is described in more detail in the accompanying *Data in Brief* article (Custodio et al., 2020). For the purpose of the current baseline study, we focused on the macronutrient content (i.e., carbohydrates, protein, and fat) and the total calories based on food composition tables (Longvah et al., 2017). All data were stored in a database (Samaddar et al., 2020).

### Statistical analysis

The content of the databases were accessed in Python programming language (Version 3.6.6) using the SQLAlchemy (Version 1.2.7) (Bayer, 2012) object relational mapper. Data were processed, and summary statistics (e.g., frequencies) and co-occurrence matrices from text corpora were generated using the pandas library (Version 0.23.0) (McKinney, 2010) and the itertools module. The dietary diversity score of each occasion in each state was determined by counting the number of dish groups (Rathnayake et al., 2012; Swindale and Bilinsky, 2006). Data visualization was performed using Matplotlib (Version 2.2.2) (Hunter, 2007) and Seaborn (Version 0.9.0) (Waskom et al., 2018) packages.

## Results and discussion

### Target population

The workshop in Kolkata was attended by nine experts while the workshop in Bhubaneswar had eight experts participating. Their expertise was well balanced among the four different professional backgrounds that we defined in our selection criteria (see Section 2.1), i.e. Nutrition, Home Science, Food Technology, and Foodservice Industry (i.e., chefs and restaurateurs). Refining the definition of the target population, i.e., low-to middle-income rural and urban households, was a crucial and complex process, requiring additional context, in understanding food choice behaviors. The experts agreed that an income class has a distinct culture, with varying aspirations and outlook that cannot be defined solely by earnings. By a similar token, diets can be seen with a politico-historical lens; i.e., diets are influenced by class, cultural, and imperial relations (Nandy, 2004). The middle class is included in the study's target population because this class embodies the poor's aspirations for upward social mobility, particularly in colonial and post-Independence India: educated, urban, and employed in white-collar jobs (Lobo and Shah, 2015). Likewise, the capital cities of Kolkata, West Bengal and Bhubaneswar, Odisha are characterized as cosmopolitan cities, with high living costs; these cities are also symbolic of the aspirations of the rest of the population of these states, especially in terms of lifestyle and food choices (Redfield and Singer, 1954). The experts, thus, concentrated on defining the target population based on the characteristics of low- and middle-income households in these cities. The experts established an upper limit for the income^3^ of the target population for each city, i.e. 1092 USD in Kolkata and 780 USD in Bhubaneswar. The definitions of low- to middle-income households, based on cut-off points, varied between the two states. These values are within the range of the annual incomes of the “strivers” subgroup of the middle-income class as defined by the National Council of Applied Economic Research, based on 2001–2002 prices (Meyer and Birdsall, 2012). However, the experts were not able to set a lower monthly income limit due to differing values from various government-developed classification schemes. According to the experts, most of the households belonging to the low-income bracket were involved in farming; it was difficult to estimate their incomes because these households typically combine subsistence and market-oriented production. Hence, the food choices of people in these income brackets were expected to be limited to the subsistence level. On the other hand, various government schemes related to minimum wage, minimum guaranteed wage days, and income per person made it difficult for experts to agree on setting the lower limit of earnings for lower income households. The upper limits sufficed for the experts to contextualize the GSR framework's hierarchy of occasions, dishes, and ingredients; thus allowing the experts to capture the diversity of food choice of the target population in the two states.

### Gastronomic system of the target population

The gastronomic system of the target population constructed by the experts in both states was captured in an online database (Ynion et al., 2020; summarized in Table S1). Below, we describe the results in more detail following the GSR framework.

**Occasions.** Occasions were defined as the culturally prescribed times in a day when people consume food for regular and special times. Food consumption occasions vary widely at the individual level and are more frequent than the three meals over a 12-h period dictated by conventional wisdom (Gupta et al., 2017). Hence, the experts identified six existing occasions both in Kolkata and Bhubaneswar: Breakfast, Morning (AM) Snack, Lunch, Afternoon (PM) Snack, Dinner, and Special Occasions (Table S1). Note that it was not necessary for every member of each household to follow all the food consumption occasions identified in their everyday life. For instance, the experts recognized that most of the dining occasions they identified are centered around consumers' work hours. Breakfast is the first occasion of food consumption in the household after the longest period of sleep; it is relatively a simple meal because it is composed of food items from at least one food group (O'Neil et al., 2014). On the other hand, lunch is the food consumption occasion that happens around the middle of the day (Davidson, 2014) while dinner is the main food consumption occasion at the end of the day (Anonymous, 2019). Note that the definitions of these occasions do not consider the actual time of consumption, considering that shift workers could have different eating schedules (Waterhouse et al., 1997). Assuming that the consumer works during the day, the snack occasion was divided by the experts into two distinct occasions: the AM Snack occurring between Breakfast and Lunch and the PM Snack occurring between Lunch and Dinner. In general, the timing of consumption of each occasion varies from household to household, from urban to rural, and also with the work profile and lifestyle nature of the household members. A study monitoring times of food intake by 94 healthy adults in Uttar Pradesh, for example, demonstrated that the subjects had a food consumption occasion roughly every 3 h and 15 min (Gupta et al., 2017), consistently accounting for the two snack occasions. Special occasions, in contrast, are not dependent on the consumers' work schedule. Rather, they vary based on the nature of festivals, celebrations, or religious rituals.

**Dishes.** A dish is a very important component through which food choices are expressed by any community and culture. In Kerala and Delhi, for example, the dish choices in a household highly depend on the preferences of the husband and the children; such prioritization was seen as a factor leading to unhealthy food choices (Bailey et al., 2018). To develop a baseline database for the diversity of food choices in West Bengal and Odisha, the experts identified 131 unique dishes that were “typical” for the food consumption occasions of the target population, excluding those consumed during special occasions (Ynion et al., 2020; summarized in Tables S1 and S2). It is interesting to note that in both states, the experts in each state elicited the lowest number of dishes for the AM Snack occasion (Table S2), implying that AM Snack may not be as popular a food consumption occasion among consumers in both states; in contrast, for example, to PM Snack which appears to have more dish options.

**Fig. 4:**
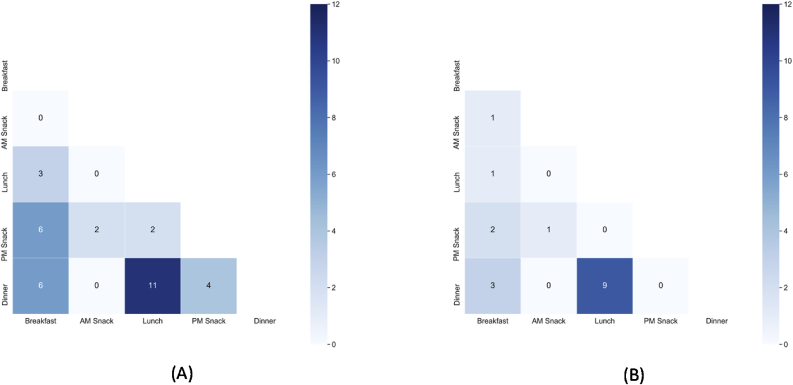
Heat maps indicating the number of common dishes between occasion pairs in (A) Odisha and in (B) West Bengal. The intensity of the color is associated with the number of common dishes. The darker the color, the less distinct the two occasions are (i.e., more dishes in common). Source: Ynion et al. (2020). (For interpretation of the references to color in this figure legend, the reader is referred to the Web version of this article.)

The number of common dishes between pairs of occasions indicates how differentiated one occasion is from the other (Fig. 4). AM Snack is the most distinct occasion, with only two dishes in common with PM Snack in Odisha (Fig. 4A) and one dish in common with PM Snack and Breakfast in West Bengal (Fig. 4B). In Odisha, Lunch and Dinner were least differentiated in that they had the highest number of common dishes, followed by Breakfast and Dinner, and Breakfast and PM Snack (Fig. 4A). Lunch and Dinner also had the highest number of common dishes in West Bengal; however, other occasion pairs appeared to have more distinct food options (Fig. 4B). Hence, Fig. 4 suggests that occasions are generally more differentiated in West Bengal than in Odisha, which validates our rationale for the application of the GSR framework and our decision to zoom in further on the state of West Bengal.

The database developed by the experts and summarized in Table S1 reveals that out of the total of 131 dishes captured in both states, almost a quarter of them (31 or 24%) were referred to by the same name in both states (excluding Special Occasion), which is quite expected due to the proximity of the two states with each other. Oriyas claim that many Bengali dishes have originated from Odisha, tracing them back to the long culinary tradition of Puri's Jagannath Temple being exported to the upper-class Bengali kitchen during the Bengal cultural renaissance in the British Raj (O'Brien, 2013). Over time, some form of food localization must have occurred because the occasions in which 14 of these common dishes are consumed differed between West Bengal and Odisha (Table S1).

Another notable characteristic of food choices in West Bengal and Odisha is evidence of influences from other food cultures. A recurring point of “contention” during the elicitation process was the definition of a “typical dish”. There was a lot of discussion among the experts regarding certain dishes that were not considered in any traditional category. Many non-traditional dishes, originating from outside the state, have become a part of the food tradition and the daily diets of people in Kolkata and Bhubaneswar. Pant (2010, p. 18) noted that Kolkata, the former capital during British colonial administration, has historically played a leading role in adopting and adapting other cultural influences, especially in its cuisine. Dosa, a crepe-like layered rice batter mentioned as a PM snack in Odisha, is believed to originate from Tamilakam (Achaya, 2000). Idli, a steamed breakfast cake elicited in both states, is believed to have originated in ancient Hindu kingdoms of Indonesia (Achaya, 2000) and is similar in form but not exactly the same as the Chinese steamed rice cake (Nandy, 2004). Biriyani, a mixed dish of rice and meat, is a product of the fusion of Persian cuisine and Hindustani food cultures that occurred in the Mughal Empire (Collingham, 2006). Chowmin, a stir-fried noodle dish mentioned as a PM snack in West Bengal, has Hakka Chinese origins but its sauces were adapted to local tastes when first introduced in Kolkata (C. S. Bhattacharya, 2015). Momo (dumpling) and thukpa (hot noodle soup) (Angchok et al., 2009), both elicited in the West Bengal workshop, originated from Tibet (Boi, 2015). The food influences of these dishes indicate that Indians in both states have borrowed heavily from other food cultures and made these dishes their own (Nandy, 2004). It was observed that the assimilation of dishes with out-of-state influences was more prominent in West Bengal than in Odisha based on the dishes elicited from the experts (Table S1). The international borders West Bengal shares with Nepal, Bangladesh, and Bhutan (De, 1990), as well as the assignment of Kolkata as a presidency town during the British colonial period (Banerjee-Guha and Sinha, 2019), has most likely contributed to the international flavor in the cuisine. Though these dishes appeared to be more representative of urban Kolkata's tastes and preferences, further discussions among the experts indicated that these dishes have become very common even in rural areas. The information elicited from the experts reflected the role played by the city in promoting cultural changes as aspirational for the rest of the state and the dynamic nature of a food culture.

The dishes for Special Occasions reflected old traditions, expectations, and aspirations of a community irrespective of its income group or class. Dishes served during Special Occasions highlight budget constraints that lead to compromises in food choices. These compromises are based on the scale and intimacy of the special occasion (e.g., marriage celebration vis-à-vis small family gathering). Thus, the “specialness” of a dish does not depend on the preparation cost. For instance, the experts noted that dishes served at a big wedding reception may consist of affordable dishes while those at a small family gathering may be more expensive.

**Fig. 5:**
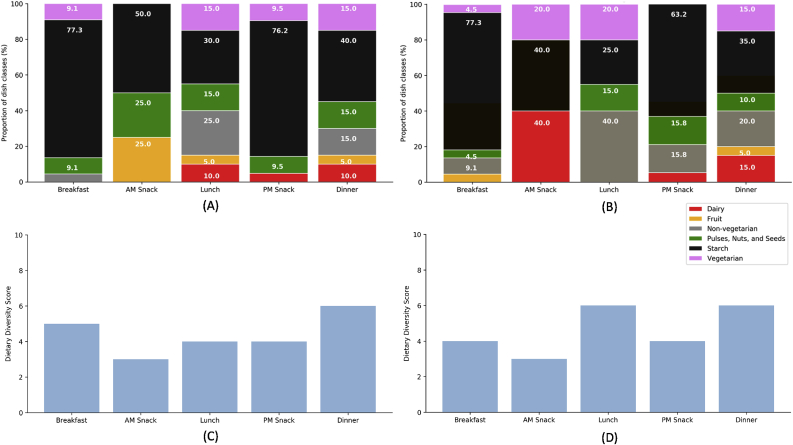
Comparison of proportions of dishes in Odisha (A) and West Bengal (B), and of food group counts for Odisha (C) and West Bengal (D), based on dish classification in the five daily dining occasions. Source: Ynion et al. (2020).

**Ingredients.** In Odisha, AM Snack was the least nutritionally diverse among the different dining occasions because only three food groups were represented (Fig. 5A and C). On the other hand, Lunch and Dinner were the most nutritionally diverse occasions for food choice because the elicited dishes represented all six food groups. Meanwhile, Breakfast and PM Snack both featured four food groups each. For all five daily occasions, the starch food group had the highest proportion of dishes identified by the experts. Non-vegetarian dishes were elicited for Breakfast, Lunch, and Dinner but not for the snack occasions. Pulses, nuts, and seeds were also represented in all five daily occasions. Dairy dishes were identified for Lunch, Dinner, and PM Snack. Vegetarian fare were identified for all occasions except AM Snack. Fruits were found in AM Snack, Lunch, and Dinner.

In West Bengal, AM Snack was also the least diverse among the dining occasions, with three food groups represented (Fig. 5B and D). Dinner was the most represented among the occasions, with six food groups identified based on the dishes elicited. Breakfast had five food groups, while Lunch and PM Snack both had four food groups. Vegetarian dishes were represented in all occasions except PM Snack. The dairy group was found in both snack occasions and in Dinner. Lunch was the only occasion in which starch-type dishes did not make the biggest proportion of dishes. Instead, non-vegetarian dishes comprised most of the dishes elicited from the experts. Non-vegetarian dishes were also found in Breakfast and Dinner. For these three occasions, non-vegetarian dishes had higher proportions than vegetarian dishes. The relative prevalence of non-vegetarian ingredients in West Bengal is in line with dietary changes associated with increasing disposable incomes and structural transformations linked with urbanization (Huang and Bouis, 1996; Mottaleb et al., 2018a, 2018b). The proximity to bodies of water has influenced the presence of fish as an ingredient in food choices in both states (Misra, 2011). Particularly among the Bengalis, eating fish is related to their identity (the Bengali description “Mache Bhaate Bangali” translates into “Fish Rice Bengali” in English); there are over 40 fish types used in Bengali cooking that are obtained solely from freshwater sources (Appadurai, 1988; Saxena, 2018). Dishes in the pulses, nuts, and seeds group were identified in all occasions except for AM Snack. Fruit dishes were elicited in AM Snack, Lunch, and Dinner.

### Nutrition interventions

The experts identified and ranked a set of possible nutrition interventions in each state (Table 1). The key nutritional concerns were the excessive use of fat/oil, refined wheat flour, and peeled vegetables, and malnutrition caused by lack of proteins, iron deficiency, and loss of nutritive value during cooking. Specific target populations included children, pregnant women, adolescent girls, and anemic people. Interventions targeted all three entry points in the GSR framework: occasions, dishes, and ingredients; which provides some validation that the experts had well understood the framework. In terms of agents of change, there was a strong focus on the role of the government, schools, activists, and NGOs. Interestingly, the private sector was not mentioned. Finally, the experts believed in a strong role of awareness campaigns in combination with programs that increase availability of nutritious food items for the target populations.

**Table 1:** Nutrition interventions proposed by the experts during the expert elicitation workshops.

State	Nutritional concern	Target population	Entry point	Action/intervention	Agents of change	Impact pathway	Rank
Odisha	High use of low-quality fat/oil which has adverse effects to health (i.e. lifestyle diseases)	Children and teenagers	Occasion level: PM Snacks Dish level: Junk food (including fast food)	Dishes like steamed food, germinated grains with nuts and oil seeds, and eggs	Ministry of Women and Child Development (WCD), dietitians, nutritionists, and mass media	Awareness through mass media (e.g., advertisements)	1
	Frequent use of refined wheat flour, peeled vegetables, faulty cooking process	All age groups in both rural and urban areas	Dish level: dishes with low fiber	Multigrain dishes and inclusion of flour with husk or peel of vegetables Wash vegetables before cutting	Household	Awareness through training, demonstration, or information and communications technology (ICT)	2
	Combat malnutrition	Children 3–6 years old	Dish level	Use of protein-rich under-utilized seeds and oil cakes	Research organizations and the government	Research organizations to develop nutritious food items and make them available to children	3
West Bengal	Undernutrition (severe acute malnutrition)	Preschool children age 6 years old and above in both rural and urban areas	Occasion level: Taking small frequent meals	High calorie and high protein dishes (e.g., suji payesh, chanar, etc.)	Integrated Child Development Services (ICDS) centers, Government, and NGOs	Awareness and monitoring	1
	Iron deficiency	Pregnant women, adolescent girls, and anemic people	Ingredient level	Inclusion of sprouted Bengal gram, green gram, and jaggery instead of sugar in diet Promotion of kitchen garden	*Panchayats* (village councils), Schools, NGOs, the Government, and ICDS	Awareness and availability of improved value-added products in shops	2
	Loss of nutritive value while cooking and handling food	Lower middle-income group	Dish	Inclusion of fruit or edible raw vegetables in diet	Social activists, *Anganwadi* workers, Accredited Social Health Activist (ASHA), Community facilitators, and School professionals	Awareness programs, feeding demonstrations, nutrition care and counselling session of mothers in *Anganwadi* Centers (AWC)	3

### Nutritional implications

While refining the list of dishes in West Bengal, the dishes were viewed as cultural representations of the dietary pattern of West Bengal and Bengalis and not as sources of nutrition. For example, rice consumption is deeply embedded in Bengali culture; hence, Bengalis eat rice as part of their social norms, rather than for rice's nutritional content.

Prediction of the total nutrient intake based on results from the expert elicitation workshops was challenging as experts prioritized and listed a maximum of 20 dishes per occasion. Many of the dishes were grouped based on commonalities during prioritization. Hence, an informal structured survey conducted as a supplement to the Kolkata workshop was conducted to expand the list of dishes based on the expert elicitation results and estimate nutritional contents of dishes consumed in all five daily occasions identified through the GSR framework (i.e., excluding Special Occasions). It should be noted that instead of treating it as an indicator of nutritional intake, the output of the informal survey was treated as an inventory of dishes from which consumers can choose what to eat for a particular occasion. This survey yielded a final database of 158 unique dish names for West Bengal and—counting variants in terms of ingredient composition—164 recipes (Samaddar et al., 2020 summarized in Table S3). Table 2 shows the number of dishes identified per occasion; it also indicated that dinner had the most recipes (i.e., dish variants) while AM Snack had the fewest.

**Table 2:** Number of dishes by occasions in West Bengal.

Occasion	Number of dishes or recipes^a^
Breakfast	62
AM Snack	41
Lunch	63
PM Snack	60
Dinner	65

a The number of dishes does not add up to 164 (the number of unique recipes) because the same recipe (i.e., dish variant) may be found on more than one occasion.

Based on Table S3, overlaps of dish names were observed among the different occasions; for instance, Lunch and Dinner had the highest number of common dishes, with 51% dish overlap. The timing between Breakfast and AM Snack is close but only 23% of the dishes were common between these two occasions; which suggests that the two occasions are distinct (i.e., AM Snack is not late Breakfast). PM Snack also proved to be a distinct eating occasion from the preceding and succeeding occasions, with only 9% and 14% dish overlap with Lunch and Dinner, respectively. Though both snacks, AM and PM Snacks were also determined to be distinct from each other, with only 23% dish overlap. On the other hand, six dishes were found in all five daily occasions: aloo bhaja, brinjal bhaja, brinjal bharta, chapati, raw rice, and steamed rice.

**Fig. 6:**
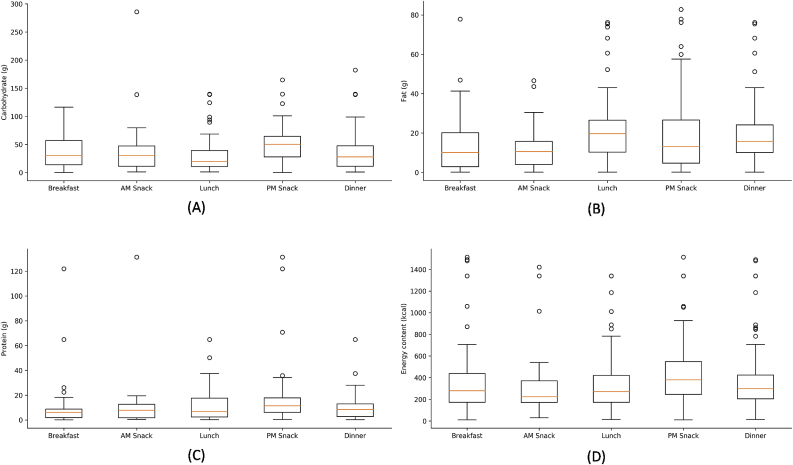
Box plots showing the distribution of carbohydrate (A), fat (B), protein (C), and energy (D) contents of dishes per occasion, based on the adult portions derived from the nutritional analyses of dishes obtained in the informal household survey conducted in Kolkata. Source: Samaddar et al. (2020).

Fig. 6 visualizes the distributions of macronutrient and energy content of the samples of dishes (based on adult portions) that were identified for each occasion. The box plots indicate the minimum (lower line), first and third quartile (box), median (red line), maximum (upper line) and outliers (dots). The carbohydrate content range was 0–286 g (Fig. 6A), indicating that Bengalis prefer a carbohydrate-rich diet. This type of diet makes them feel satiated throughout the day (Chambers et al., 2015; Zhang et al., 2018). The median carbohydrate content of the dish options was highest in PM Snack and lowest in Lunch. On the other hand, fat content of the dishes, across all occasions, was estimated at 0.06 g–83 g (Fig. 6B). Such range in fat content suggests that quite a number of dishes identified in these occasions are fried and oily. It was noted that Breakfast had the lowest median for fat content while Lunch had the highest. The protein content of the dishes ranged between 0.04 g and 131 g (Fig. 6C). Similar to carbohydrate content, the median protein content of dish options was highest for PM Snack; however, it was lowest for Breakfast. PM Snack had the highest median for energy content and AM Snack had the lowest (Fig. 6D).

The trend of relatively high carbohydrate and energy contents in food options for PM Snack, compared to the other occasions suggest that consumers may feel the physiological need to top up with an energy-dense snack (e.g., carbohydrate-rich food) in the afternoon after consuming lower energy- and carbohydrate-content of food options from Breakfast to Lunch. The food options during PM Snack could be characterized as energy-dense, nutrient-poor, and may contain high contents of sugar, fat, and salt (reviewed in Hess et al., 2017). Though the median fat content for PM Snack food options was not consistent with the definition of a “snack food”, its range was notably largest for PM Snack (Fig. 6B). Consumption of food high in sugar, fat, and sodium tends to occur without hunger cues and to be influenced by social and environmental factors; as a result, snacking may be associated with increased body weight and poor nutrition (reviewed in Bellisle, 2014; Hess et al., 2017).

Inferences can be made on this diet pattern and the physiological changes that may accompany it based on the dishes that define each eating occasion. For example, consumption of fat-rich dishes in large quantities can lead to obesity, cardiovascular problems, and other non-communicable diseases (Virtanen et al., 2014; Xu et al., 2006). Additionally, consumption of starchy and heavy dishes coupled with fried food probably contributes to increasing cases of obesity, diabetes, and cardiovascular disease among the Bengali population (Barik et al., 2016).

## Conclusion

Among the tools available in the GSR framework (Cuevas et al., 2021), expert elicitation proved to be a powerful tool for developing a preliminary understanding and context of food choice and consumption within a state, a culture, or a region, creating huge potential for future food choice studies. By engaging experts (i.e., chefs and restaurateurs, nutritionists, and home scientists) deeply embedded in the food culture of each state, each expert elicitation workshop was able to create a baseline database of food choices specifically for low- and middle-income households in Odisha and West Bengal in eastern India. Understanding and identifying the traditional food choices and changing food patterns was challenging given the heterogeneity of food culture and diversity within different regions and communities within the two states. It became clear, during the course of the workshops, that determining the food choices of the middle class in the capital cities—Bhubaneswar, Odisha and Kolkata, West Bengal—was crucial to understanding the social aspirations of low-income households in these states. Logistical and resource constraints did not allow multiple workshops in various regions, which would have enabled capturing more heterogeneity in the gastronomic system.

Following the hierarchical nature of the GSR framework (i.e., occasions, dishes, ingredients), the experts were able to put the food choices of the target populations into cultural and socioeconomic context. They captured daily food choice of the target population in eastern India through a database of 131 unique dishes associated with five differentiated daily dining occasions. The database reflects a combination of traditional and non-traditional dishes, indicating changing preferences of food habits across urban and rural contexts. Classifying the dishes according to their main ingredient provided indications of dietary diversity per occasion. Results indicate that AM Snack had the least diverse dining options in both states; while the most diverse dining options occurred at Dinner in Odisha, and Lunch and Dinner in West Bengal. This information provides a first glimpse of the nutrition diversity of the different occasions, which may be used in designing nutrition interventions that go beyond focusing on ingredients and dishes and are based on better planning of eating occasions (Kahleova et al., 2017; Kant, 2018; Mattes, 2018; Parr et al., 2018; Raynor et al., 2018). The experts identified possible nutrition interventions in each state targeting all three entry points in the GSR framework and emphasizing the role of awareness campaigns.

The West Bengal food choices were supplemented with macronutrient information, providing nutritional implications about these food choices for the target population. Results suggest that the Bengali gastronomic system features a lot of carbohydrate- and energy-dense dishes, and that many of these dishes are fried and oily. Such food options could be associated with the rise of occurrence of non-communicable lifestyle diseases such as diabetes mellitus type II, obesity, and cardiovascular diseases.

Expert elicitation is, hence, a useful first step in capturing diversity of food choice of a target population and identifying nutrition interventions that can nudge consumers towards planetary health diets. The gastronomic system mapped by the experts provides a first glimpse of the potentially unhealthy occasions, dishes and ingredients in eastern India. While this baseline uncovers cultural drivers and diversity of food choice, it does not reflect actual behavior; the gastronomic system needs to be validated through consumer surveys in a second stage. For example, the occasions, dishes, and ingredients can be included in a survey aiming at estimating frequencies of the occurrence of eating occasions, dishes, and ingredient pairings. The latter allows for early identification of unhealthy ingredients or dishes, or planning of occasions that occur frequently according to consumers' stated food choice behavior. In the same surveys, consumers’ knowledge of and attitudes towards nutrition interventions can be gauged, providing a first glimpse of the acceptability of these interventions, how consumers would integrate these changes into their diets and what the predicted nutritional implications would be. Consumer behavior can be further elicited and tested through behavioral experiments. The hierarchical system of occasions, dishes, and ingredients identified through expert elicitation in this study can, for example, be incorporated in interactive tablet applications (e.g., Demont et al., 2019), which enable testing which nutrition interventions are most effective in nudging Indian households towards healthier rice-based diets.

The gastronomic system captured in the database through expert elicitation provides a useful baseline for nutritionists, policy makers and food system actors designing nutrition intervention strategies with the aim of catalyzing dietary changes towards planetary health diets. The multi-layered and hierarchical structure of the gastronomic system enables designing nutrition intervention strategies at three different levels or entry points. At the most basic level, interventions can target ingredients or types of food (e.g., fruits and vegetables) and focus on improving the balance of nutrients (e.g., macro- and micronutrients) by encouraging the consumption of healthy ingredients, discouraging the consumption of unhealthy ingredients, and promoting diet diversity through ingredient diversity. At the dish level, interventions can target dishes and dish preparations techniques by encouraging the consumption of healthy dishes, discouraging the consumption of unhealthy dishes, and promoting diet diversity through dish diversity. Finally, interventions can target the occasion level by encouraging healthy eating behavior and discouraging unhealthy eating behavior, based on better planning of eating occasions (e.g., “Breakfast like a king, lunch like a prince and dine like a pauper”) or introducing new occasions (e.g., healthy AM or PM snack, based on fruits or vegetables). A shift towards planetary health diets will require a change in general nutrition education and “local interpretation and adaptation that reflects the culture, geography, demography of the population, and individuals” (Willett et al., 2019b, p. 10). Through the GSR framework, the experts provided rich contextual information on the local food culture in eastern India, including the nutritive value of commonly consumed dishes. Nutritionists, policy makers, and food system actors can simultaneously target all three entry points in their nutrition intervention programs to nudge households towards planetary health diets while preserving the rich cultural heritage on which their gastronomic system is based.

## Funding sources

This research was funded by the 10.13039/501100015815CGIAR Program on Rice and the Drivers of Food Choice (DFC) Competitive Grants Program (Grant no. OPP1110043). The DFC Competitive Grants Program is funded by the UK Government's Department for International Development and the Bill & Melinda Gates Foundation, and is managed by the University of South Carolina, Arnold School of Public Health, USA. However, the views expressed in this article do not necessarily reflect the UK Government's official policies.

## CRediT authorship contribution statement

**Arindam Samaddar:** Methodology, Investigation, Resources, Writing - original draft, Writing - review & editing, Project administration. **Rosa Paula Cuevas:** Conceptualization, Methodology, Software, Formal analysis, Investigation, Data curation, Writing - original draft, Writing - review & editing, Visualization. **Marie Claire Custodio:** Methodology, Investigation, Data curation, Writing - review & editing, Project administration. **Jhoanne Ynion:** Methodology, Investigation, Data curation, Writing - review & editing, Project administration. **Anindita Ray (Chakravarti):** Formal analysis, Investigation, Resources, Data curation, Writing - review & editing, Project administration. **Suva Kanta Mohanty:** Investigation, Resources, Writing - review & editing, Project administration. **Matty Demont:** Conceptualization, Methodology, Validation, Formal analysis, Investigation, Resources, Writing - review & editing, Visualization, Supervision, Funding acquisition.

## Declaration of competing interest

The authors declare that they have no known competing financial interests or personal relationships which have, or could be perceived to have, influenced the work reported in this article.

## Footnotes

2 Gastronomy is often narrowly interpreted as an expensive form of food appreciation that solely matters to the urban elite. However, in the GSR framework, gastronomy is defined as “an appreciation and understanding of the many avenues of cooking and food production” including food quality, foodways, and culinary anthropology (Katz and Weaver, 2003, p. 102; cited in Cuevas et al., 2017), which suggests that it matters to all income classes. Secondly, as Banerjee and Duflo (2007) noted, the pleasure derived from food consumption may matter more to the poor than its nutritional value. Finally, even if preferences for gastronomy are more pronounced among the urban elite, the rest of the population tends to develop aspirations for urban lifestyle and food choices over time due to the cultural role of cities (Redfield and Singer, 1954).

3 Values based on the average conversion rate of 64.0807 INR to 1 USD on July 31, 2017, rounded to the nearest whole number (https://www.poundsterlinglive.com/best-exchange-rates/us-dollar-to-indian-rupee-exchange-rate-on-2017-07-31).
