# Supplementary material for: Capturing diversity and cultural drivers of food choice in eastern India
Source: Int J Gastron Food Sci. 2020 Dec;22:100249. doi: 10.1016/j.ijgfs.2020.100249 (PMC7737094; doi:10.1016/j.ijgfs.2020.100249)
Supplement: Multimedia component 2 [file mmc2.doc]

**Table S1**

List of dishes and their eating occasions based on expert elicitation workshops conducted in West Bengal (indicated by black circles) and in Odisha (indicated by white triangles).

| **Dish name** | **Occasion** | | | | | |
| --- | --- | --- | --- | --- | --- | --- |
|  | **B** | **AM** | **L** | **PM** | **D** | **SO** |
| Aloo chop (telethaja) |  |  |  | ⬤ |  |  |
| Aloo dum |  |  |  | ▽ |  |  |
| Aloo paratha |  |  |  | ⬤ |  |  |
| Aloo posto |  |  | ⬤ |  |  |  |
| Aloo tikia |  |  |  | ⬤ |  |  |
| Aloo sabji | ▽ |  |  |  | ⬤ |  |
| Bamboo shoot |  |  | ⬤ |  |  |  |
| Bari curry |  |  | ⬤ |  |  |  |
| Biryani |  |  |  |  |  | ▽ |
| Biscuit | ⬤ | ▽ |  | ▽ |  |  |
| Bread | ⬤ |  |  |  |  |  |
| Bread pakoda |  |  |  | ▽ |  |  |
| Cake | ⬤ | ⬤ |  |  |  |  |
| Chakuli | ▽ |  |  | ▽ | ▽ |  |
| Chhanna |  | ⬤ |  |  |  |  |
| Chapati | ▽⬤ |  | ▽⬤ |  | ▽⬤ |  |
| Chapati+milk | ⬤ |  |  |  |  |  |
| Chat |  |  |  | ▽ |  |  |
| Chattu | ▽⬤ |  |  |  |  |  |
| Chena payas |  |  |  |  |  | ▽ |
| Chhole bhature |  |  |  |  |  | ▽ |
| Chicken kassa |  |  |  |  |  | ⬤ |
| Chidwas pulau |  |  |  | ⬤ |  |  |
| Chili mushroom |  |  |  |  |  | ▽ |
| Chowmin (veg) |  |  |  | ⬤ |  |  |
| Chuda | ⬤ |  |  |  |  |  |
| Chuda bhaja |  |  |  | ▽ |  |  |
| Chuda chakta | ▽ |  |  |  |  |  |
| Chuda santula | ▽ |  |  | ▽ |  |  |
| Chutney |  |  |  |  | ▽⬤ |  |
| Corn flakes | ▽⬤ |  |  |  |  |  |
| Crab curry |  |  |  |  |  | ▽ |
| Curd |  | ⬤ |  |  |  |  |
| Dahi vada |  |  |  | ▽ |  |  |
| Dal |  |  | ▽ |  | ⬤ |  |
| Dal with vegetables |  |  |  |  | ⬤ |  |
| Dalia upma | ▽ |  |  |  |  |  |
| Dalma |  |  | ▽ |  | ▽ |  |
| Dhoka |  |  | ⬤ |  |  |  |
| Dhokar dalna |  |  |  |  |  | ⬤ |
| Dosa masala |  |  |  | ▽ |  |  |
| Egg | ⬤ |  |  |  |  |  |
| Egg curry |  |  | ▽⬤ |  | ▽⬤ |  |
| Egg roll |  |  |  | ⬤ |  |  |
| Fish head and vegetables |  |  | ⬤ |  |  |  |
| Fish head dal |  |  | ⬤ |  |  |  |
| French toast |  |  |  | ⬤ |  |  |
| Fried fish |  |  |  |  |  | ⬤ |
| Fried rice |  |  |  |  |  | ▽ |
| Fried vegetables |  |  | ▽⬤ |  | ▽⬤ | ⬤ |
| Fruit | ⬤ | ▽ |  |  |  |  |
| Geri-googly curry |  |  | ⬤ |  |  |  |
| Ghugni | ▽⬤ |  | ▽ | ▽⬤ | ▽ |  |
| Gola roti | ⬤ |  |  |  |  |  |
| Hilsa bhappa |  |  |  |  |  | ⬤ |
| Ice cream |  |  |  |  |  | ⬤ |
| Idli | ▽⬤ |  |  |  |  |  |
| Jilabi | ⬤ |  |  |  |  |  |
| Kachkalar kofta |  |  |  |  |  | ⬤ |
| Khatta |  |  | ▽ |  |  |  |
| Khichdi |  |  | ▽⬤ |  | ⬤ | ▽⬤ |
| Luchi | ▽⬤ |  |  |  | ⬤ | ▽⬤ |
| Macher chop |  |  |  | ⬤ |  |  |
| Macher jhol |  |  | ▽⬤ |  | ▽⬤ |  |
| Madhi chakta | ▽ |  |  |  |  |  |
| Maka sijha |  |  |  | ▽ |  |  |
| Mangsheer jhol (mutton) |  |  | ▽⬤ |  | ⬤ | ▽ |
| Mashed potato |  |  | ▽⬤ |  |  |  |
| Mix vegetables |  |  | ▽⬤ |  | ▽ | ▽ |
| Momo |  |  |  | ⬤ |  |  |
| Moori masala |  | ▽⬤ |  | ▽⬤ |  |  |
| Motor paneer |  |  |  |  |  | ⬤ |
| Mudhi khira | ⬤ |  |  | ▽ |  |  |
| Murgir jhol |  |  | ▽⬤ |  | ▽⬤ | ▽ |
| Mushroom curry |  |  |  |  |  | ▽ |
| Mutton biryani |  |  |  |  |  | ⬤ |
| Mutton kasa |  |  |  |  |  | ⬤ |
| Nan |  |  |  |  |  | ⬤ |
| Noodles |  |  |  | ▽⬤ |  |  |
| Omelet | ▽ |  |  |  |  |  |
| Pakhala bhath | ▽ |  | ▽ | ▽ | ▽ |  |
| Palak paneer |  |  |  |  |  | ▽ |
| Paneer curry |  |  | ▽ |  | ▽⬤ | ▽ |
| Panipuri |  |  |  | ▽⬤ |  |  |
| Pantha bhath | ⬤ |  |  |  |  |  |
| Papad |  |  | ▽ | ⬤ |  |  |
| Paratha | ⬤ |  |  |  | ▽ | ▽ |
| Payesh |  |  |  |  | ⬤ | ▽⬤ |
| Potoler dorma |  |  |  |  |  | ⬤ |
| Prawn curry |  |  | ▽ |  |  | ▽ |
| Prawn malaikari |  |  |  |  |  | ⬤ |
| Puffed rice+water | ⬤ |  |  |  |  |  |
| Pulao |  |  |  |  | ⬤ | ⬤ |
| Ragi porridge | ▽ |  |  |  | ▽ |  |
| Raita |  |  | ▽ |  | ⬤ |  |
| Rajma curry |  |  |  |  | ▽ | ▽ |
| Rice |  |  | ▽⬤ |  | ▽⬤ |  |
| Rice pitha |  |  |  |  | ▽ |  |
| Roasted peanut |  | ▽ |  | ⬤ |  |  |
| Rosogollah |  |  |  |  | ⬤ | ▽ |
| Saag |  |  | ▽⬤ |  |  |  |
| Salad |  |  |  |  | ▽ | ▽⬤ |
| Sambar | ▽ |  |  |  |  |  |
| Samosa |  |  |  | ▽ |  |  |
| Sandesh |  |  |  | ⬤ |  | ⬤ |
| Sandwich | ▽ |  |  |  |  |  |
| Sandwich (non-veg) |  |  |  | ⬤ |  |  |
| Santula | ▽ |  |  |  |  |  |
| Simei kheer |  |  |  | ▽ | ▽ | ▽ |
| Sooji halwa | ▽⬤ |  |  | ⬤ |  |  |
| Sooji upma | ▽ |  |  | ▽ |  |  |
| Soya bean curry |  |  | ⬤ |  | ⬤ |  |
| Sprouts |  | ⬤ |  |  |  |  |
| Sukuti |  |  | ⬤ |  |  |  |
| Sweets |  |  |  |  | ▽ |  |
| Thukpa | ⬤ |  |  |  |  |  |
| Uttapam |  |  |  | ⬤ |  |  |
| Vada | ▽ |  |  | ▽ |  |  |
| Vegetable curry | ⬤ |  |  |  | ⬤ |  |
| Vegetable pakoda |  |  |  | ▽ |  |  |

Source: Ynion et al. (2020)

**Table S2**

Number of dishes elicited during the expert elicitation workshops conducted in West Bengal and Odisha, based on Table S1.

| **Occasion** | **Number of Dishes** | | |
| --- | --- | --- | --- |
|  | **West Bengal** | **Odisha** | **Total** |
| Breakfast | 22 | 22 | 44 |
| AM Snacks | 5 | 4 | 9 |
| Lunch | 20 | 20 | 40 |
| PM Snacks | 19 | 21 | 40 |
| Dinner | 20 | 20 | 40 |
| Special Occasions | 19 | 21 | 40 |
| Total number of dishes (all occasions) | 105 | 108 | 213 |
| Total number of dishes (special occasion excluded) | 86 | 87 | 173 |
| Total number of unique dishes (no duplicates; special occasions excluded) | 70 | 61 | 131 |

Source: Ynion et al. (2020)

**Table S3**

Dishes commonly consumed in West Bengal and estimated nutritional content (carbohydrate, protein, fat, and energy) of one adult serving portion.

| **Recipe No.** | **Dish name** ^a^ | **Occasion** ^b^ | **Serving size (g)** | **Carbo-hydrate (g)** | **Protein (g)** | **Fat (g)** | **Energy (kcal)** |
| --- | --- | --- | --- | --- | --- | --- | --- |
| 1 | Aloo barbati fry | B | 100 | 9.09 | 1.08 | 10.09 | 131.55 |
| 2 | Aloo bhaja | D | 70 | 11.30 | 0.80 | 20.05 | 228.50 |
| 3 | Aloo bhaja | B, A, L, P | 45 | 6.78 | 0.48 | 15.03 | 164.10 |
| 4 | Aloo bhate | B, L | 37 | 6.78 | 0.48 | 7.03 | 92.10 |
| 5 | Aloo chokha | B | 168 | 32.83 | 5.69 | 6.36 | 480.64 |
| 6 | Aloo chop | P | 90 | 14.70 | 1.34 | 20.11 | 232.00 |
| 7 | Aloo dum | B, L, D | 102 | 20.39 | 2.58 | 20.98 | 267.40 |
| 8 | Aloo kabli | A, P | 320 | 73.16 | 131.37 | 4.26 | 394.75 |
| 9 | Aloo papaya curry | L | 134 | 17.77 | 2.31 | 10.50 | 172.10 |
| 10 | Aloo paratha | B, P, D | 113 | 56.36 | 7.24 | 10.58 | 349.54 |
| 11 | Aloo posto | L | 120 | 18.64 | 7.18 | 21.08 | 378.20 |
| 12 | Aloo tikiya | P | 145 | 20.09 | 2.09 | 5.30 | 405.40 |
| 13 | Amaranth fry | L | 85 | 5.55 | 2.10 | 10.30 | 123.00 |
| 14 | Banana | B, A | 75 | 27.20 | 1.20 | 0.30 | 116.00 |
| 15 | Bean and radish | D | 55 | 2.76 | 0.62 | 5.06 | 85.25 |
| 16 | Bengal gram dal | D | 119 | 34.72 | 12.47 | 17.64 | 347.19 |
| 17 | Bhel puri | P | 95 | 39.31 | 4.16 | 3.16 | 201.88 |
| 18 | Chicken biryani | L, D | 318 | 89.62 | 27.12 | 19.47 | 638.29 |
| 19 | Mutton biryani | L, D | 468 | 98.66 | 37.45 | 43.03 | 850.09 |
| 20 | Bitter gourd fry | B, A, L, D | 40 | 1.26 | 0.48 | 10.06 | 97.50 |
| 21 | Boiled eggs | B | 50 | 0.00 | 6.65 | 6.65 | 86.50 |
| 22 | Boiled pulse | L, D | 40 | 23.60 | 10.04 | 0.28 | 137.20 |
| 23 | Boiled whole Bengal gram | B, A, D | 50 | 30.45 | 8.55 | 2.65 | 180.00 |
| 24 | Bori curry | L | 145 | 66.61 | 27.28 | 22.60 | 579.35 |
| 25 | Sandwich (non-veg) | P | 175 | 38.94 | 17.76 | 4.84 | 259.40 |
| 26 | Brinjal bhaja | B, A, L, P, D | 105 | 6.27 | 1.30 | 30.46 | 304.25 |
| 27 | Brinjal bharta | B, A, L, P, D | 119 | 11.32 | 1.67 | 15.56 | 191.95 |
| 28 | Butterscotch pastry | L, D | 395 | 138.69 | 21.40 | 60.62 | 1186.05 |
| 29 | Cabbage curry | L, D | 105 | 3.62 | 1.74 | 17.37 | 149.40 |
| 30 | Cauliflower curry | B, L, D | 115 | 6.14 | 2.74 | 21.10 | 225.50 |
| 31 | Cauliflower pakoda | P | 275 | 89.75 | 34.15 | 36.80 | 808.50 |
| 32 | Chaler payesh | D | 320 | 75.49 | 16.06 | 10.18 | 479.60 |
| 33 | Chana | B | 50 | 30.45 | 8.55 | 2.65 | 180.00 |
| 34 | Chana | D | 60 | 36.54 | 10.26 | 3.18 | 216.00 |
| 35 | Chanachur | A, P | 75 | 36.40 | 12.90 | 23.60 | 195.00 |
| 36 | Chapati | B, A, L, P, D | 100 | 64.17 | 10.57 | 1.53 | 1340.00 |
| 37 | Chatu gola | B, A | 85 | 44.68 | 16.88 | 3.99 | 282.45 |
| 38 | Chicken momo with thukpa | P | 377 | 90.29 | 33.48 | 3.93 | 520.09 |
| 39 | Chicken tandoori | L | 249 | 26.49 | 29.43 | 24.16 | 409.95 |
| 40 | Chidwa polao | P | 155 | 59.87 | 5.38 | 0.81 | 267.90 |
| 41 | Chili oil | B | 7 | 0.18 | 0.04 | 5.01 | 45.80 |
| 42 | Chili chicken | D | 172 | 23.52 | 21.46 | 21.50 | 386.16 |
| 43 | Chocolate pastry | L, P, D | 415 | 139.37 | 23.85 | 76.18 | 1339.65 |
| 44 | Chutney | B, L, D | 50 | 27.09 | 0.24 | 0.42 | 111.55 |
| 45 | Chutney | L | 215 | 124.25 | 1.47 | 0.32 | 506.05 |
| 46 | Corn flakes | B, A | 100 | 30.50 | 5.70 | 0.40 | 146.00 |
| 47 | Cottage cheese | A | 100 | 1.20 | 18.30 | 20.80 | 265.00 |
| 48 | Cucumber | B, P | 75 | 1.88 | 0.30 | 0.075 | 9.75 |
| 49 | Chicken curry (murgir jhol) | L, D | 186 | 13.90 | 22.02 | 18.77 | 308.67 |
| 50 | Cutlet (kabab) | P | 250 | 49.06 | 20.41 | 60.00 | 818.00 |
| 51 | Dahivada | P | 360 | 27.52 | 70.80 | 82.80 | 793.50 |
| 52 | Dal kachori | B, P | 255 | 54.10 | 121.90 | 77.91 | 1059.45 |
| 53 | Dal pakoda | L | 54 | 13.36 | 5.30 | 20.18 | 256.42 |
| 54 | Dalia khichdi | B, L, D | 245 | 68.42 | 64.92 | 16.77 | 706.85 |
| 55 | Dhokla | P | 160 | 65.30 | 26.42 | 25.85 | 600.75 |
| 56 | Dry Bombay duck | L, D | 200 | 27.94 | 4.70 | 27.76 | 396.65 |
| 57 | Egg toast | B | 130 | 25.95 | 12.00 | 28.57 | 411.10 |
| 58 | Egg curry | A, L, D | 110 | 3.21 | 19.44 | 22.32 | 258.45 |
| 59 | Poached egg (oil) | B, P | 55 | 0.00 | 6.65 | 11.65 | 131.50 |
| 60 | Poached egg (water) | B, P | 50 | 0.00 | 6.65 | 6.65 | 86.50 |
| 61 | Egg roll | P | 180 | 58.14 | 15.22 | 22.36 | 495.10 |
| 62 | Fish chop | P | 255 | 53.17 | 24.90 | 29.74 | 553.35 |
| 63 | Fish finger | P | 275 | 35.50 | 19.62 | 57.56 | 708.65 |
| 64 | Fried fish | P | 140 | 5.82 | 13.44 | 15.79 | 219.30 |
| 65 | Fish head dal | L | 225 | 37.14 | 20.38 | 41.26 | 601.85 |
| 66 | Fish head vegetables | L | 293 | 22.90 | 10.65 | 26.14 | 369.61 |
| 67 | Fish kachori | D | 200 | 75.60 | 20.70 | 51.20 | 846.50 |
| 68 | French toast | P | 100 | 22.74 | 7.46 | 14.32 | 249.60 |
| 69 | Fried dhoka | L | 185 | 57.32 | 29.38 | 73.83 | 1010.40 |
| 70 | Fried lal saak | L, D | 90 | 7.04 | 2.42 | 10.30 | 130.25 |
| 71 | Fried peanuts | A, P | 30 | 8.01 | 7.86 | 11.94 | 171.00 |
| 72 | Fried rice | B, L, D | 233 | 57.13 | 18.19 | 30.72 | 578.58 |
| 73 | Fruit salad | L | 230 | 28.93 | 2.11 | 0.55 | 129.20 |
| 74 | Gajar halwa | B | 183 | 30.17 | 4.72 | 46.96 | 562.13 |
| 75 | Ghoogni | B, P | 125 | 33.93 | 11.35 | 16.38 | 328.80 |
| 76 | Gola roti | B | 80 | 41.64 | 7.26 | 21.02 | 384.60 |
| 77 | Guava | A | 100 | 11.20 | 0.90 | 0.30 | 51.00 |
| 78 | Gup chup | P | 270 | 122.62 | 21.12 | 52.92 | 1050.95 |
| 79 | Gur | B, D | 100 | 84.87 | 1.85 | 0.16 | 1480.00 |
| 80 | Idli with sambar and coconut chutney | B | 265 | 83.94 | 22.39 | 23.74 | 642.45 |
| 81 | Jalebi | B, P | 155 | 62.15 | 26.15 | 3.64 | 383.00 |
| 82 | Keema curry | D | 105 | 12.27 | 2.10 | 40.72 | 424.30 |
| 83 | Kellogg's chocos with milk | A | 300 | 39.30 | 12.10 | 8.60 | 280.00 |
| 84 | Khichdi | A, L, P, D | 125 | 57.45 | 11.48 | 10.63 | 371.25 |
| 85 | Khirer chop | A | 600 | 285.90 | 15.60 | 23.65 | 1422.00 |
| 86 | Chicken korma | L | 281 | 30.42 | 31.74 | 52.25 | 727.75 |
| 87 | Litti | P | 230 | 67.95 | 17.32 | 63.96 | 926.05 |
| 88 | Luchi | B, P, D | 100 | 59.12 | 8.80 | 20.72 | 458.40 |
| 89 | Maggi | B, A, P | 100 | 57.70 | 11.90 | 18.70 | 446.00 |
| 90 | Malpoa | B | 390 | 116.14 | 8.80 | 41.28 | 870.80 |
| 91 | Mango murabba | P | 265 | 164.84 | 3.08 | 1.03 | 680.90 |
| 92 | Mango pickle | B, L, D | 60 | 12.46 | 0.17 | 25.02 | 275.80 |
| 93 | Marie biscuit | B, A, P | 100 | 79.34 | 6.60 | 5.54 | 393.80 |
| 94 | Masala dosa | B, P | 236 | 81.96 | 15.40 | 34.01 | 638.36 |
| 95 | Masoor dal | B | 50 | 23.00 | 8.78 | 11.22 | 228.15 |
| 96 | Masoor dal | L, D | 60 | 28.90 | 11.29 | 11.28 | 262.45 |
| 97 | Misti doi | L, D | 140 | 44.40 | 3.20 | 4.10 | 226.20 |
| 98 | Mixed pickle | L, D | 175 | 22.67 | 3.28 | 75.48 | 783.40 |
| 99 | Mixed vegetable curry | B, L, D | 207 | 17.92 | 3.12 | 30.38 | 357.23 |
| 100 | Mixed vegetables | B, L, D | 207 | 17.92 | 3.12 | 30.38 | 357.23 |
| 101 | Momo | P | 345 | 100.79 | 35.77 | 31.79 | 838.35 |
| 102 | Moori masala | A | 108 | 43.21 | 4.35 | 5.10 | 223.27 |
| 103 | Mung dal | B, A,L, D | 58 | 30.43 | 12.19 | 5.80 | 222.47 |
| 104 | Mutton curry | L, D | 367 | 47.63 | 22.26 | 68.20 | 888.22 |
| 105 | Mutton liver curry | L, D | 189 | 17.90 | 12.92 | 32.15 | 409.76 |
| 106 | Thukpa | B | 160 | 44.94 | 5.01 | 22.82 | 405.18 |
| 107 | Roasted peanut | P | 50 | 13.35 | 13.20 | 19.90 | 285.00 |
| 108 | Omelet | L, D | 82 | 1.72 | 6.89 | 21.68 | 229.58 |
| 109 | Onion pakoda | A, P | 175 | 51.92 | 17.80 | 28.98 | 539.25 |
| 110 | Orange | B, A | 60 | 6.54 | 0.42 | 0.12 | 28.80 |
| 111 | Brinjal pakoda | P | 145 | 35.16 | 13.82 | 39.25 | 547.10 |
| 112 | Palak paneer | L | 200 | 13.88 | 17.40 | 30.82 | 388.29 |
| 113 | Potato paneer | L | 240 | 21.78 | 20.88 | 31.72 | 455.55 |
| 114 | Paneer curry | D | 75 | 0.90 | 13.72 | 15.60 | 198.75 |
| 115 | Panta rice | B, D | 100 | 78.24 | 7.94 | 0.52 | 1491.00 |
| 116 | Fried papad | A, L | 25 | 11.80 | 5.02 | 5.14 | 113.60 |
| 117 | Papri chat | P | 265 | 57.08 | 8.67 | 3.36 | 292.80 |
| 118 | Paratha | B, A, P, D | 73 | 47.32 | 6.60 | 10.54 | 310.74 |
| 119 | Plain dosa | P | 110 | 63.46 | 13.00 | 20.48 | 491.80 |
| 120 | Polao | B | 150 | 63.46 | 5.56 | 15.43 | 415.50 |
| 121 | Posto bata | L, D | 40 | 11.04 | 6.51 | 15.79 | 212.40 |
| 122 | Roasted potato | B | 105 | 14.89 | 1.54 | 4.28 | 328.45 |
| 123 | Potato chips | A | 50 | 11.82 | 0.97 | 15.80 | 181.84 |
| 124 | Potato chop with puffed rice | B | 85 | 43.58 | 4.23 | 5.08 | 236.60 |
| 125 | Potato curry | B | 70 | 13.56 | 0.96 | 10.06 | 148.20 |
| 126 | Potato ladies finger curry | B, L, D | 120 | 15.14 | 1.94 | 10.17 | 159.50 |
| 127 | Potato raw banana curry | L | 125 | 24.70 | 1.75 | 15.25 | 242.70 |
| 128 | Potato ivy gourd curry | B, A, L, D | 95 | 10.26 | 1.20 | 15.11 | 181.60 |
| 129 | Puffed rice | B, P | 100 | 77.68 | 7.47 | 1.62 | 1514.00 |
| 130 | Radish potato curry | A | 100 | 16.89 | 1.46 | 15.68 | 199.55 |
| 131 | Raita | L, D | 138 | 39.18 | 2.43 | 2.34 | 102.87 |
| 132 | Rajma | A, P, D | 110 | 37.84 | 12.67 | 10.78 | 299.20 |
| 133 | Rasgolla | D | 110 | 40.84 | 12.81 | 14.56 | 344.70 |
| 134 | Raw rice | B, A, L, P, D | 50 | 39.50 | 3.20 | 0.26 | 173.00 |
| 135 | Steamed rice | B, A, L, P, D | 50 | 39.50 | 3.20 | 0.26 | 173.00 |
| 136 | Rice papad | P | 25 | 19.56 | 6.25 | 0.13 | 372.15 |
| 137 | Rohu fish | L, D | 133 | 12.25 | 12.77 | 24.13 | 285.77 |
| 138 | Rohu fish curry | A, L, D | 133 | 12.25 | 12.77 | 24.13 | 285.77 |
| 139 | Rohu fish fry | D | 80 | 4.97 | 7.76 | 15.66 | 191.95 |
| 140 | Saag | L | 85 | 93.75 | 50.25 | 22.75 | 780.00 |
| 141 | Salad | L, D | 40 | 2.78 | 0.34 | 0.06 | 13.10 |
| 142 | Samosa | B, A, P | 135 | 79.55 | 11.40 | 10.92 | 462.25 |
| 143 | Sandesh | B, A, P, D | 177 | 30.64 | 12.57 | 14.54 | 303.58 |
| 144 | Semolina halwa | B, P, D | 135 | 30.40 | 5.92 | 6.58 | 204.20 |
| 145 | Semolina upma | P | 115 | 51.96 | 8.96 | 14.54 | 375.77 |
| 146 | Simay payesh | D | 360 | 88.36 | 21.66 | 26.13 | 654.50 |
| 147 | Soaked whole Bengal gram | B | 50 | 30.45 | 8.55 | 2.65 | 180.00 |
| 148 | Soya bean curry | L, D | 65 | 8.98 | 11.41 | 19.94 | 261.10 |
| 149 | Sukuti | L | 108 | 8.18 | 10.82 | 10.41 | 167.90 |
| 150 | Sweet pancake | A | 240 | 138.75 | 9.78 | 46.60 | 1014.00 |
| 151 | Tangra fish curry | L, D | 155 | 7.14 | 8.31 | 25.58 | 292.20 |
| 152 | Tarka | D | 110 | 34.08 | 13.01 | 15.78 | 330.05 |
| 153 | Thukpa | P | 140 | 36.18 | 6.08 | 0.71 | 177.50 |
| 154 | Uttapam with sambar and coconut chutney | P | 109 | 51.96 | 4.21 | 10.28 | 317.81 |
| 155 | Vanilla cake | A, P | 215 | 77.20 | 18.72 | 9.30 | 467.45 |
| 156 | Vanilla ice cream | A, P | 85 | 21.00 | 3.80 | 43.65 | 481.70 |
| 157 | Sprouts | A | 50 | 28.35 | 12.00 | 0.65 | 167.00 |
| 158 | Vegetable chow mein | B, P | 325 | 94.99 | 14.76 | 1.24 | 450.45 |
| 159 | Vegetable chow mein | D | 575 | 182.32 | 28.07 | 2.37 | 863.60 |
| 160 | Vegetable pasta | B, L, P | 150 | 28.00 | 5.48 | 11.18 | 235.60 |
| 161 | Vegetable sandwich | B | 165 | 46.44 | 6.21 | 0.55 | 215.30 |
| 162 | Vegetable soup | A | 55 | 17.17 | 1.76 | 0.32 | 144.05 |
| 163 | Vetki fish curry with cauliflower | L, D | 175 | 18.44 | 10.88 | 21.58 | 298.55 |
| 164 | Yoghurt | A, L | 100 | 4.40 | 3.20 | 4.10 | 67.00 |

^a^ The dishes were identified through separate informal structured surveys conducted by the Department of Food & Nutrition, Maharani Kasiswari College, Calcutta University.

^b^ The letters denote the occasions as follows: B = breakfast; A = AM snack; L = lunch; P = PM snack; and D = dinner.

Source: Samaddar et al. (2020)
